# Evaluating complex interventions and health technologies using normalization process theory: development of a simplified approach and web-enabled toolkit

**DOI:** 10.1186/1472-6963-11-245

**Published:** 2011-09-30

**Authors:** Carl R May, Tracy Finch, Luciana Ballini, Anne MacFarlane, Frances Mair, Elizabeth Murray, Shaun Treweek, Tim Rapley

**Affiliations:** 1 Faculty of Health Sciences, University of Southampton, UK; 2 Institute of Health and Society, Newcastle University, UK; 3 Agenzia Sanitaria e Sociale Regionale, Bologna, Italy; 4 Discipline of General Practice, National University of Ireland, Galway, Ireland; 5 Academic Unit of General Practice and Primary Care, Institute of Health and Wellbeing, University of Glasgow, UK; 6 Research Department of Primary Care and Population Health, University College London, UK; 7 Quality, Safety & Informatics Research Group, University of Dundee, UK

## Abstract

**Background:**

Normalization Process Theory (NPT) can be used to explain implementation processes in health care relating to new technologies and complex interventions. This paper describes the processes by which we developed a simplified version of NPT for use by clinicians, managers, and policy makers, and which could be embedded in a web-enabled toolkit and on-line users manual.

**Methods:**

Between 2006 and 2010 we undertook four tasks. (i) We presented NPT to potential and actual users in multiple workshops, seminars, and presentations. (ii) Using what we discovered from these meetings, we decided to create a simplified set of statements and explanations expressing core constructs of the theory (iii) We circulated these statements to a criterion sample of 60 researchers, clinicians and others, using SurveyMonkey to collect qualitative textual data about their criticisms of the statements. (iv) We then reconstructed the statements and explanations to meet users' criticisms, embedded them in a web-enabled toolkit, and beta tested this 'in the wild'.

**Results:**

On-line data collection was effective: over a four week period 50/60 participants responded using SurveyMonkey (40/60) or direct phone and email contact (10/60). An additional nine responses were received from people who had been sent the SurveyMonkey form by other respondents. Beta testing of the web enabled toolkit produced 13 responses, from 327 visits to http://www.normalizationprocess.org. Qualitative analysis of both sets of responses showed a high level of support for the statements but also showed that some statements poorly expressed their underlying constructs or overlapped with others. These were rewritten to take account of users' criticisms and then embedded in a web-enabled toolkit. As a result we were able translate the core constructs into a simplified set of statements that could be utilized by non-experts.

**Conclusion:**

Normalization Process Theory has been developed through transparent procedures at each stage of its life. The theory has been shown to be sufficiently robust to merit formal testing. This project has provided a user friendly version of NPT that can be embedded in a web-enabled toolkit and used as a heuristic device to think through implementation and integration problems.

## Background

Recent years have seen steadily more sophisticated approaches to the evaluation of complex interventions and technological innovations in health care. In particular, evaluation frameworks like that proposed by the UK Medical Research Council have emphasized the need to understand the complex components and contingent underpinnings of outcomes studies, especially clinical trials [1,2]. At the same time, there have been calls for theory-driven approaches to such work [3,4]. Theories are valuable in such work not because they provide clear and unambiguous solutions to outcomes problems, but because they can provide robust, generic, and transferable explanations of the processes that shape these outcomes. They perform the further useful function of making transparent the assumptions of researchers and others that underpin research questions, methodology, and explanations [5,6].

Normalization Process Theory (NPT) [7], and its predecessor, the Normalization Process Model [8,9] provides a conceptual framework to assist in understanding and explaining the dynamic processes that are encountered during the implementation of complex interventions and technological or organizational innovations in healthcare.

Robust social science theories already explain some important features of implementation and integration processes: individual differences in attitudes and intentions in relation to new technologies and practices (e.g. Theory of Planned Behavior [10]), the flow of innovations through social networks (e.g. Diffusion of Innovations Theory [11]), and reciprocal interactions between people and artifacts (e.g. Actor Network Theory [12]). NPT differs from these theories because it offers an explanatory model of the routine embedding of a classification, artefact, technique or organizational practice in everyday work. NPT focuses on the agentic contribution--the things that people do--of individuals and groups. It thus explains phenomena not well covered by existing theories.

NPT was initially developed as an applied theoretical model to assist clinicians and researchers to understand and evaluate the factors that promote and inhibit the routine incorporation of complex healthcare interventions in practice. It started from a set of empirical generalizations derived from secondary analyses of qualitative data collected in a wide variety of studies of complex interventions in healthcare. This resulted in the original constructs of the model [8]. The further empirical applications of the model showed that while it could explain factors that promote and inhibit collective action, how participants came to engage and support the practice and how they reflected on and evaluated it remained unexplained. Through the development of further constructs, accounting for how people make sense of a practice, participate in it and appraise what they do, the model became a theory. Over the past four years it has been developed as a middle-range theory of socio-technical change [7], which characterizes the mechanisms involved in the embedding of social practices within their immediate and broader social contexts.

The starting point of NPT is that to understand the embedding of a practice we must look at what people actually do and how they work [7]. NPT focuses on four theoretical constructs, which characterize mechanisms that are energized by investments made by participants.

(i) Processes of individual and communal sense making that promote or inhibit the coherence of a complex intervention to its users. These processes are driven by investments of meaning made by participants.

(ii) Processes of cognitive participation that promote or inhibit users' enrolment and legitimation of a complex intervention. These processes are driven by investments of commitment made by participants.

(iii) Processes of collective action that promote or inhibit the enacting of a complex intervention by its users. These processes are driven by investments of effort made by participants.

(iv) Processes of individual and communal reflexive monitoring that promote or inhibit users' comprehension of the effects of a complex intervention. These processes are driven by investments in appraisal made by participants.

These mechanisms, and their underpinning investments, are constrained (and released) by the operation of norms (notions of how beliefs, behaviours, and actions should be accomplished); and conventions (how beliefs, behaviours, and actions are practically accomplished). In this context, mechanisms, investments, and constraints form processes of organized, dynamic, and contingent interaction between: agents (the individuals or groups that interact in encounters around a practice); objects (the classifications, artifacts, practices and procedures employed by agents); and contexts (the technical and organizational structures in which agents and objects are implicated) [8,9]. The primary focus of NPT is therefore the analysis of social action. As far as possible, its central constructs and their dimensions refer to observable social mechanisms [13-15] that shape the practical workability and integration of some complex intervention or technology. For health services researchers interested in process evaluation NPT provides a verifiable and empirically grounded model of the operation of factors that promote and inhibit the routine incorporation of interventions in everyday practice. For social scientists, NPT provides a well characterized middle-range theory of socio-technical change.

Although it is a relatively new theory, it has been used to:

• inform the development and evaluation of complex clinical and organizational interventions for mental health care [16,17]

• examine the work processes entailed in implementing treatment regimes into patients' routines [18]

• inform evaluations of treatment modalities in cancer [19], and diabetes [20].

• aid understanding of the findings of randomised controlled trials for psychosocial distress and nurse-led clinics for heart failure treatment [21], chronic constipation [22] and collaborative care for depression [23]

• inform the redesign of primary care mental health services [24] and self-management training packages [25].

• support the development and application of decision-support tools [26] and inform a systematic review of evidence about their utilization [27]

• aid understanding of the implementation of telecare and e-health systems in a wide variety of contexts [28-38]

Theories of all kinds are formed through complex interpretive processes that lead to inherently abstract products. Abstraction is, in fact, a necessary condition of a theory, since it must be sufficiently context-independent to be applicable to the range of relevant cases that it might be required to explain [39]. The problem that users of a theory face, then, is translating the theory from its abstract context-independent form into a form that can be used to solve problems in everyday settings. NPT is no exception. Our aim in the work reported here, therefore, has been to translate NPT's constructs into a set of statements that can be used by managers, clinicians, and researchers to work through problems of design and implementation in relation to complex interventions and new health technologies. These simplified constructs were translated into a set of statements that form the basis of a toolkit http://www.normalizationprocess.org for clinicians, managers and policy-makers interested in utilizing NPT in their work.

The purpose of this simplification work was to develop a set of generic statements that could be configured as the 'front end' of a web enabled toolkit for users of NPT. For this reason, we sought engagement and critique from NPT's user communities (Health Services Researchers, Clinical Researchers, and Social Scientists). The co-production of theories is normal in large scale investigations in the natural sciences but is much less common in the social and behavioural sciences. In such circumstances, peers are usually asked to test theories rather than collaborate in defining the means by which they are operationalized. We have sought to be as transparent as possible in the generation of the theory, and as inclusive as possible in its operationalization and stabilization in practice. Our view is that this continuous 'road testing' of basic constructs and components of the theory has done more than ensure construct validity. It has ensured that the theory is relevant to its users. In this paper we present a simplified set of 16 statements that express key elements of NPT but which can be applied without a detailed knowledge of the underlying theory. However, we must also offer a caveat. Our objective in this work was to simplify a set of theoretical constructs for heuristic purposes, and not to develop a set of validated questions that could be immediately embedded in quantitative research instruments or qualitative interview schedules. The purpose of this paper is to make transparent the process by which the 16 statements and explanations were generated, and thus be clear about the foundation of the claims we make about them.

## Methods

### Understanding how NPT was applied by users to real-world problems

Prior to the idea for the toolkit emerging, we sought to better understand the ways that potential users of NPT could apply it to real world problems. Between 2006 and 2009 we engaged with multiple potential users.

Engaging potential users included presentations to researchers and practitioners that linked NPT's core constructs to practical research and development problems. It also included open workshops and master-classes for researchers and practitioners interested in NPT in the UK, Australia, Canada, and the US, in addition to individual correspondence and discussion with both experienced senior and neophyte researchers interested in employing NPT in their work. These encounters provided us with an opportunity to explore the views of NPT's potential users and their critiques of both its core assumptions and constructs and of the ways that these were presented. Some potential users were sceptical, arguing that NPT offered no advantage over the Theory of Planned Behavior [10] because its predictive value was unknown, and others that it was incompatible with Actor-Network Theory [12] because of its insistence on explanation over description.

At the same time, we closely engaged with critical actual users of NPT. This included work to stabilize the constructs of the theory that we have described elsewhere [40], apply them in practice to statement development for surveys, systematic reviews and qualitative investigations [41,42] and to define appropriate ways to apply the theory. We did this through the medium of meetings of a Peer Learning Set funded by the UK National Institute of Health Research, and personal communications with researchers using NPT in existing studies [17,23,25,26,34]. We used the group of actual users to help identify the sources of ambiguity and complexity in users' experiences of the theory. It is through our engagement with these actual users that the idea of simplifying the abstract constructs and developing a tool kit first emerged.

### Translating abstract constructs into simple statements

Our second task - and the topic of this paper - was to translate the abstract constructs of the theory into their simplest possible statements, drawing, in part, on the experience we gained during the process of presenting NPT to potential and actual users. This is a process analogous, but not identical, to statement development in questionnaire design, and it rests on rigorous construct validation. We divided it into three sequential tasks.

▪ We distilled each construct to a single statement of no more than two sentences. These identified the underlying social mechanism (Coherence, Collective Action, etc), explained what factors this mechanism shaped (sense-making, enacting, etc), and specified the social investments that energized it (meaning, effort, etc). This led to four construct explanations.

▪ We met as a group and spent two days reducing each of the components of the four constructs to a single sentence that described what people do when they act in relation to them. This led to 16 component explanations.

▪ We then constructed a set of 16 statements that expressed each component as a single context-independent statement that could be addressed to participants in an implementation-integration process. This led to 16 component statements.

These statements and explanations were 'road tested' in seminars at the Mayo Clinic (Rochester, Minnesota, US) and Dundee University (Scotland, UK) in April and May 2010. On 1 June 2010 we sent the statements and explanation (See Additional File 1, first column, 'Original Statement and Construct Explanation'). Participants in this process were selected according to criterion sampling. One of us (CRM) had kept an archive of NPT related emails and other correspondence since 2004 and this formed the sampling frame from which participants were selected. The sampling criterion was that participants appeared to be sufficiently familiar with NPT to comment on attempts to translate its constructs into plain language. We invited 60 researchers to take part and they belonged to four categories: Medicine (n = 18), Nursing and Midwifery (n = 16), Professions Allied to Medicine (n = 3), and Health Services Research and Social Science (n = 23).

Respondents were asked to feedback using an on-line pro forma composed of a series of open ended questions constructed using SurveyMonkey™ (a proprietary on-line survey tool), and described in Additional File 2. The duration of this exercise was 21 days. We also invited members of the criterion sample to snowball the on-line form to members of their research groups and to other interested colleagues. Participants were asked to identify themselves by name and email address so that we could distinguish between those recruited directly and those who had copies forwarded to them as part of the snowball. We sent a single email reminder on 8 June 2010.

Data collected in this process took the form of short free text entries typed directly into the survey monkey pro-forma by respondents. Free text entries consisted of specific comments about items and statements, and more broadly focused comments about what respondents understood the value and limits of the toolkit to be. The comments about items and statements were extracted and then aggregated according to the item to which they referred in a matrix, or framework [43]. This provided a basis for subsequent work to improve the clarity and fidelity of each statement. We treated the comments about the value and limits of the toolkit as attributive statements and analysed them using a simple and descriptive thematic analysis [44].

### Road testing the web-enabled tool

The final component of this work was to embed improved and edited statements and explanations into a web-enabled tool (available at http://www.normalizationprocess.org between August 2010 and July 2013) and to invite users to apply the tool in practice and comment on it. We already had some experience of designing web-enabled tools [45]. We released the web-enabled tool on 26 July 2010, sending a URL link and invitation to researchers who had responded to our earlier on-line questionnaire, and inviting them to snowball the URL to interested colleagues. 0 We also made a single announcement on Twitter.com and CRM's personal web-page at academia.edu, again for the purposes of snowballing.

**Figure 1:**
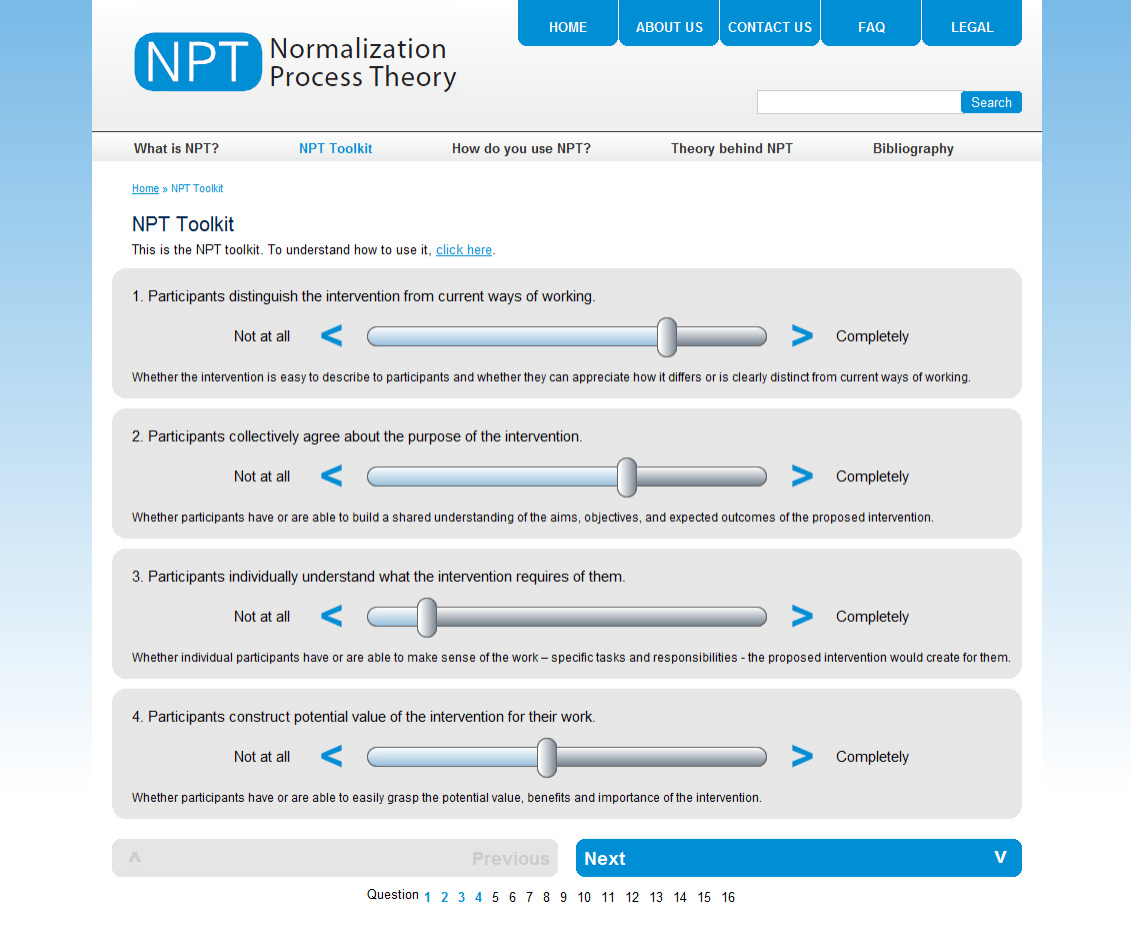
**NPT Toolkit - Web-interface - Sliding Toolbar**.

**Figure 2:**
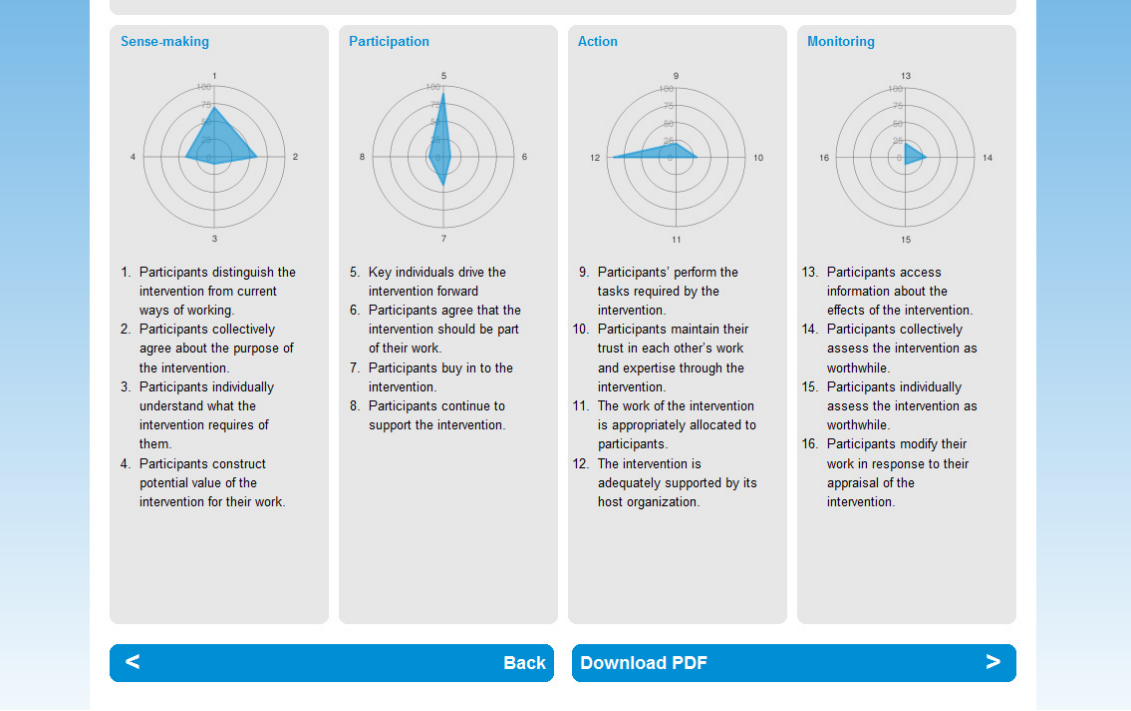
**NPT Toolkit - Reporting page - Individual Radar Plots for each Construct**.

Participants in this phase of our work were asked to work through an implementation problem using slide bars to give a subjective score to each of the statements embedded in it (an example of these, see Figure 1), and to interpret the results of this work through a set of radar plots (see Figure 2). One of us (CRM) also field-tested tested the tool with 30 participants at a meeting at the Faculty of Health and Social Development, University of Victoria, British Columbia, on 29 and 30 July 2010 to work through two implementation problems, a falls prevention initiative, and the development of a large collaborative project between the University and the Vancouver Island Health Authority.

## Results

### Responses

As Table 1 shows, we emailed a criterion sample of 60 researchers, and achieved a response of 50/60 between 1 and 21 June 2010. In addition to our criterion sample, we received responses from nine other 'snowball' respondents. Of the 10 members of the criterion sample that did not respond, four were away on sabbatical or other leave. We have no information about six other non-respondents. Of the criterion sample, 10/50 communicated their views about statements and explanations by email or telephone to CRM. Only one member of this group provided a detailed critique of the statements and explanations. The remainder made general comments about their focus and orientation. The majority of data we received was derived from 40 criterion sample respondents, and the nine snowball sample respondents who replied using the Survey Monkey tool. We have combined responses from these two groups for qualitative analysis. Table 2 describes the structure and geographical distribution of the combined study group.

**Table 1:** Purposive Sample of Respondents: Statement development phase

Response Category	Non-respondents	Respondents
Out of office auto-reply Sabbatical or other leave	5	0

Did not respond	5	

Responded using on-line pro forma		40

Responded by email or telephone		10

**Total criterion sample**	**10/60**	**50/60**

Additional snowball respondents	**N/A**	9

**Total all respondents**		**59**

**Table 2:** Professional structure of combined criterion and snowball samples: Statement development phase

	Europe	N America	Australasia	Total
**Postgraduate Student**	3	1	0	4

**Assistant Prof/**	8	5	4	17

**Lecturer/Research Fellow Associate Prof/Senior Lecturer**	4	5	3	12

**Full Professor**	13	2	2	17

**Non-academic** Practitioner*	6	3	0	9

**Total**	**34**	**16**	**9**	**59**

* Includes three senior nurses, two public health specialists, one senior informatician, and one senior civil servant.

Respondents using the SurveyMonkey pro forma asserted that they were familiar with NPT. Only 12 suggested that they possessed a low level of familiarity with the theory. We asked participants to read the statements and their explanations and to work through them in relation to an implementation practice or research problem. These respondents applied NPT to a wide variety of problems. Not all respondents provided sufficient information to identify these, but we could identify problems related to Primary Care (n = 14), Hospital Medicine (n = 7), Nursing and Midwifery (n = 6), Health Informatics (n = 5), Social Care (n = 4), and Public Health (n = 3). Ten respondents identified themselves as already using NPT as a basis for ongoing studies, and six were, or had been, involved in designing studies in which NPT was integral but which were not yet operational. In at least five of these cases, this work was accomplished in groups. A further 23 respondents said that they had reviewed the statements and their explanations through the medium of thought experiments about potential or actual implementation projects. A small number of respondents told us about the time committed to this task. This ranged from 20 minutes to three hours.

The web-enabled tool had been released for testing in a way that maximized commentary from real users. We embedded Google Analytics html code in the website and this enabled us to obtain some limited data about its usage and users. During the pilot period (26 July -26 August 2010) the website attracted 327 visits (139 new visitors and 188 return visitors) and details of these are given in Table 3. Time on site ranged from 21 to 0 minutes (mean was 4.15 minutes), and page views ranged from 21 to one (mean was 5.11). From 139 new visitors we received some 15 detailed comments on their experience of the site, using free text boxes that users could fill in as they worked through the site.

**Table 3:** Visits to http://www.normalizationprocess.org: beta testing phase

Origin	New	Returning
Direct from URL	96	157

Twitter.com	1	2

Academia.edu	2	16

Google search	13	7

Wikipedia	15	1

Yahoo search	1	-

Harvard Business	10	2

Review Blog RSS		

Mayo Clinic Intranet	1	4

### The on-line survey

All but three participants were supportive of the approach we had taken and about the statements presented to them. Many made enthusiastic comments about this, and remarked that the statements improved the workability of NPT in practice. This was especially so amongst those without a background in the social sciences. We had invited respondents to be critical, however, and most had important and useful comments to make. These took two forms. First, many respondents offered specific criticisms about the statements and their explanations. These are grouped and described in Additional File 1 (see second column, 'Users' Critique'). They related to three main kinds of problem: ambiguously worded statements and explanations; overlap, where some statements and their explanations appeared to cover the same ground as others; and dissonance, where some statements and their explanations appeared to express different concepts. As we have noted, most respondents were very positive about the statements and explanations. A medical researcher told us that:

It provided food for thought about the issues involved in trying to bring together a team of both researchers and practitioners to design and implement an intervention. In particular it helped me to understand that the reasons why we are having so much difficulty is that the research team themselves do not have a shared view and understanding of what the intervention is we are trying to implement and this is contributing to our problems in engaging the primary care partners in the project.

A nurse researcher told us that:

The questions serve as an inventory; anticipatory guidance before embarking on a change in practice or as a reflective/evaluation tool. In my example, the intervention was introduced to the inter-disciplinary [team] as a 'pilot'. I was asked to assist with evaluating the 'pilot'. If these 16-questions would have been available I could envision utilizing them as a guide for evaluation focus groups/interviews with end-users.

In these contexts, respondents seemed to be using the statements and their explanations in exactly the way we had intended them - as sensitizing tools, heuristic devices, to support thinking through an implementation task. Importantly, though, we did not intend these statements to be used as the basis for specific research instruments or as verbatim statements for an interview schedule.

Beyond this, respondents offered interesting and useful general critiques that often made wider methodological points. One health services researcher wrote that:

[I] can see why it is seductive. I imagine some of it might work for trial interventions where you have a clear comparator - e.g. differentiate the intervention from usual practice (our 'intervention' is the work now and we do not really have a comparator as such). It looks helpfully simple (so will appeal to many because of this) - not too long - easy to read - etc but then using it, it unravels and seems less useful (I feel a bit the way I did the first time I used the SF36 in a face to face interview - I ended up wanting to qualify every answer)

This reflects the central problem with the process of translation and simplification. It reduces the potential for acknowledging complexity within the tool. But there is a further problem here which is the extent to which a small number of respondents saw themselves reading something that was analogous to a structured research instrument rather than a set of statements that were intended to sensitize users to process problems in implementation. Complexity was added, too, by the use of theoretical vocabulary within the explanations and beyond. Another respondent wrote that:

I felt some of the language was still too technical. I would not use your technical descriptions "differentiation" etc - just ... complicate the understanding of the concept by using words which could be interpreted as having a different meaning to the one expressed in the question. Specific examples: 3 "make sense of the work" - would understand better as "make sense of what they had to do" (and work in 7) 8 "define the actions and procedures" - perhaps "define what needs to be done" 9 "enact the intervention" - perhaps "carry out the intervention" 10 see above 14 and 15 - I prefer "think it is worthwhile" or "agree about the worth of the effects" - it is the phrase "worth of the effects" which feels a little foreign.

While for others it was:

A little tricky to work with at times. The terms don't always appear to coincide with the descriptions provided. Sometimes it was helpful to simply ignore the term, and concentrate on the description. Furthermore, the bolded "headline" doesn't always convey what is indicated in the explanation below it.

Several respondents remarked on the problem of seeking to integrate understanding the statements and their explanations at a more general level.

I am not sure if having 2 bits of text i.e. question and description for each question might confuse some people (as I have had this mentioned to me at a conference when I did something similar) although personally I do feel it helps the users understanding and quite like it.

Once again, these problems stem from the process of reduction and editing that led to the construction of the statements and their explanations. A small number of respondents sought to suggest solutions to such problems. For example:

It might be best to have a two part question with an amplification of the question in the second part. For example, "participants can/could discover the effects of the intervention", for example "from formal or informal evaluation". Also the "questions" are not phrased as questions but as statements - would be better as questions.

The qualitative analysis that we present here is a simple and descriptive one. Data was in the form of free text entries in an on-line pro-forma. Respondents invested a good deal of effort in working through the statements and their explanations. As we have seen, they identified problems that were about meaning (focusing on the content of statements and their explanations), and about structure (focusing on the relationship between individual statements and their explanations).

### Responses to the web enabled tool

We received a small number of electronic and in-person responses to the web enabled tool. Most of these were congratulatory. One respondent - a sociologist - felt that the web-enabled tool over-simplified NPT and meant that it would be difficult to interpret. Two respondents pointed to continuing difficulties with continued ambiguity or overlap for statements 2 & 14, 3 & 15, 5 & 11, 6 & 7. To solve this problem we amended these items again. Other users sought more advice about how to solve implementation problems, and a reduction in 'jargon'. For one user, however, the result was clarity and workability:

Love it, at least I can understand it now. All I need to remember is SPAM (sense-making, participation, action, monitoring). This will be a great tool to map progress.

Despite the undesirable mnemonic 'SPAM', this was the result that we were aiming for.

### Final set of statements

The key result of this process was a set of statements that expressed in the simplest possible terms the components of the four constructs of Normalization Process Theory, and that could be applied in practice as heuristic tools implementation and evaluation problems. The final set of statements produced through this process was:

1. participants **distinguish **the intervention from current ways of working

2. participants **collectively agree **about the purpose of the intervention

3. participants **individually understand **what the intervention requires of them

4. participants **construct potential value **of the intervention for their work

5. **Key individuals drive **the intervention forward

6. participants **agree **that the intervention should be part of their work

7. participants **buy into **the intervention

8. participants **continue to support **the intervention

9. participants' **perform the tasks **required by the intervention

10. participants **maintain their trust **in each other's work and expertise through the intervention

11. the work of the intervention is **allocated appropriately **to participants

12. the intervention is **adequately supported **by its host organization

13. participants **access information **about the effects of the intervention

14. participants **collectively assess **the intervention as worthwhile

15. participants **individually assess **the intervention as worthwhile

16. participants **modify their work **in response to their appraisal of the intervention

## Discussion

Respondents' critical comments on statements and explanations, as we have noted, were important and useful. We learned much about how the statements and their explanations were read and understood by a purposive international sample of researchers and practitioners. While respondents were enthusiastic and supportive about the statements and explanations, and valued the translation work that they represented, they also provided criticisms that focused our attention on problems in the way that the theory was understood when it was simplified in this way. This left us with three problems to solve.

First of all it was clear that we needed to rephrase individual statements to make their meanings clear, and to reduce problems of 'fine distinction' and overlap that affected some of them - especially in relation to statements 2, 3, 14 and 15. In fact, we rewrote almost all of the statements, working not only to clarify their meanings but also their purpose as heuristic devices to help users think through implementation processes rather than measure them. This involved producing and then choosing - by means of a simple vote by each member of the project team - alternative forms of words for each statement, and where necessary the explanation. We then undertook a final amendment phase to make them workable. The progression from original statement and explanation - through respondents' criticisms, alternative wording, voting choice, and to final version - is shown in detail in Additional File 1. We repeated this process after users had responded to the web-enabled tool. This led to the final set of statements.

The second problem was whether to do additional work to marry statements and their explanations more effectively, or whether to remove the explanations themselves. Some respondents had made a strong case for removing the explanations on the grounds that they would confuse novice users or distract expert ones. In this context, we also had to take account of the usability of the statement and explanation in the on-line toolkit. The combination of these factors led us to decide to include explanations on the web-interface (see Figure 1), and on its reports (see Figure 2). They are also embedded elsewhere in the on-line Users' Manual for NPT, where they are linked to more detailed accounts of the theory's constructs.

Finally, and rather less importantly, we had to decide whether or not to acknowledge the specific theoretical origin of each statement by assigning it the name of the component of NPT to which it referred. We chose to drop these from the toolkit. However, they remain elsewhere in the on-line User's Manual. The limitations of this study are that our sample may be biased towards a favourable view of NPT by virtue of their previously expressed interest in NPT and earlier personal contacts. A second limitation is that is it also biased towards respondents working in some capacity in academia over those working as full-time practitioners. As such, the practitioner group is relatively small and this may have implications on the potential usability of the tool for this group. Clearly, irrespective of researcher enthusiasm, practitioners, managers and policy makers, alongside patients and careers, are central to the successful embedding of interventions. However, we should note that many of these academics also had commitments as clinical practitioners, healthcare managers, and policy makers. A third limitation is that limitations on time and resources did not permit us at this stage in the project to perform cognitive interviews in which users of the statements worked through them while thinking out loud. Overall, using email and a web based tool to collect qualitative (textual) data from a purposive sample of international researchers and practitioners was highly successful, with a very small number of non-respondents. The 59 researchers and practitioners who responded to our qualitative data collection tool, and the 13 who commented on the beta version of the toolkit at http://www.normalizationprocess.org were supportive and helpful, and consistently provided us with valuable critical comments.

## Conclusions

The funding program that supported the work described in this paper was intended to support the translation of social science research into products that would have value for the wider polity. Our aim in this paper is to show how we worked towards this objective. Our aim for the project itself was to take the core constructs and components of a sociological theory and translate them into the simplest possible set of statements. These statements were designed to be used as heuristic devices in an on-line toolkit for users of the theory, and not to define questions that could be used as the basis of an instrument to measure variables derived from NPT's constructs and their components. As a result of this work we have been able to develop a simplified set of statements and explanations that translate a sociological theory into a 'user friendly' form of words. This is an important step in crossing the translational gap between the complex language of academic expert communities and the multiple everyday needs of researchers and practitioners in applied settings [46].

## Conflict of interests

CRM led the program of theory building underpinning work described here, and all authors have contributed to the development of NPT.

## Authors' contributions

All authors have contributed practically and intellectually to the work that led to this paper and have commented and agreed on the manuscript. CRM led the study. TR led the development of the web-enabled tool.

## Pre-publication history

The pre-publication history for this paper can be accessed here:

http://www.biomedcentral.com/1472-6963/11/245/prepub

## Supplementary Material

Additional file 1 **Progression from Original Statement and Explanation to beta testing phase**. Table in landscape format. Click here for file

Additional file 2 **Qualitative Data Collection Using On-Line. Pro Forma - Questions Asked**. List of questions asked on online survey. Click here for file
