# Supplementary material for: Evaluating complex interventions and health technologies using normalization process theory: development of a simplified approach and web-enabled toolkit
Source: BMC Health Serv Res. 2011 Sep 30;11:245. doi: 10.1186/1472-6963-11-245 (PMC3205031; doi:10.1186/1472-6963-11-245)
Supplement: Additional file 1 — Progression from Original Statement and Explanation to beta testing phase. Table in landscape format. [file 1472-6963-11-245-S1.PDF]

Additional File 1: Progression from Original Statement and Explanation to beta testing phase

Original Statement and Explanation	Users' Critique	Alternative Statements	Chosen Statements	Version for beta testing
1. Can/Did participants distinguish the [intervention] from existing practice?<br><br>**Differentiation:** Whether the [intervention] is easy to describe to participants and whether they can appreciate how it differs or is clearly distinct from current ways of working.	(a) not clear what 'existing practice' meant;<br>(b) whether the intervention is easy to describe to participants and whether they can appreciate how it differs or is clearly distinct from current ways of working	A1.1 Participants can/could distinguish the [intervention] from existing practice<br>A1.2 Participants can/could distinguish the [intervention] from current ways of working existing practice<br>A1.3 Participants can/could distinguish the [intervention] from existing work practices<br>A1.4 Participants can/could distinguish the [intervention] from current ways of working	1. Participants can/could distinguish the [intervention] from current ways of working<br><br>.	**1. Participants distinguish the [intervention] from current ways of working**<br><br>**Differentiation:** Whether the [intervention] is easy to describe to participants and whether they can appreciate how it differs or is clearly distinct from current ways of working.

2. Can/Did participants develop a shared agreement about the purpose of the [intervention]?<br><br>**Communal specification:** Whether participants have or are able to build a shared understanding of the aims, objectives, and expected outcomes of the proposed [intervention].	(a) not clear how shared agreement would be manifested;<br>(b) perceived overlap with statements14 and 15.	**A**2.1 Participants can/could work together to agree how to co-ordinate the [intervention]<br>**A**2.2 Participants can/could collectively agree about the purpose of the [intervention]<br>**A.2.3** Participants can\could develop a collective agreement about the purpose of the intervention	2. Participants can/could collectively agree about the purpose of the [intervention]	**2. Participants collectively agree about the purpose of the [intervention]**<br><br>**Communal specification:** Whether participants have or are able to build a shared understanding of the aims, objectives, and expected outcomes of the proposed [intervention].

3. Can/Did participants develop an understanding of what the [intervention] requires of them?<br><br>**Individual specification:** Whether individual participants have or are able to make sense of the work – specific tasks and responsibilities – the proposed [intervention] would create for them.	N/A	**A**3.1 Participants can/could individually develop an understanding of what the [intervention] requires of them?	3. Participants can/could individually develop an understanding of what the [intervention] requires of them?	**3. Participants individually understand what the [intervention] requires of them**<br><br>**Individual specification:** Whether individual participants have or are able to make sense of the work – specific tasks and responsibilities – the proposed [intervention] would create for them.

4. Can/Did participants develop a sense of the worth of the [intervention]?<br><br>**Internalization:** Whether participants have or are able to easily grasp the potential value, benefits and importance of the [intervention].	(a) not clear how wider benefits relate to personal worth;<br>(b) perceived overlap with statements13-15.	**A**4.1 Participants can/could develop a sense of the potential value of the [intervention]<br>A4.2 Participants can/could develop a sense of the potential value of the [intervention] for them/their work<br>A4.3 Participants can\could individually see the potential values of the intervention.	4. Participants can/could develop a sense of the potential value of the [intervention] for them/their work	**4. Participants construct potential value of the [intervention] for their work**<br><br>**Internalization:** Whether participants have or are able to easily grasp the potential value, benefits and importance of the [intervention].

5. Do/Did participants drive the [intervention] forward?<br><br>**Initiation**: Whether or not key individuals are able and willing to get others involved in the new practice.	(a) not clear who ‘carries forward’; (b) not clear whether initiation is about collective ability to enrol, or individual champions; (c) explanation does not fit with statement .	**A**5.1 Participants can/could <u>work together to operationalize</u> the [intervention]<br>A5.2 Participants can/could work together to operationalize (implement?) the [intervention]<br>A5.3 Participants are/could be enthused by key inidividuals to work together to operationalize/implement the [intervention]<br>**A**5.4 Participants can/could work together to initiate the [intervention]<br>A5.5 There are\were key individuals able to drive the intervention forward	5. There are\were key individuals able to drive the [intervention] forward	**5. Key individuals drive the [intervention] forward**<br><br>**Initiation**: Whether or not key individuals are able and willing to get others involved in the new practice.

6. Do/Did participants see that they are the right people to be involved in the [intervention]?<br><br>**Legitimation:** Whether or not participants believe it is right for them to be involved, and that they can make a valid contribution	(a) not clear what valid contribution means;<br>(b) may overlap with statements7 and 11.	**A**6.1 Participants can/could agree that the [intervention] is the right thing to do.<br>**A6.2** Participants see\saw that they are the right people to be involved in the intervention	6. Participants see\saw that they are the right people to be involved in the intervention	**6. Participants agree that the [intervention] should be part of their work**<br><br>**Legitimation:** Whether or not participants believe it is right for them to be involved, and that they can make a valid contribution

7. Do/Did participants buy in to the [intervention]?<br><br>**Enrolment:** The capacity and willingness of participants to organize themselves in order to collectively contribute to the work involved in the new practice.	(a) Not clear whether 'buy in' investing the effort in planning and getting the intervention off the ground, or actually delivering the intervention;<br>(b) not clear whether participants need to 'buy in' to whole or part of the intervention;<br>(c) 'buy in' not sufficiently differentiated from 'confidence';<br>(d) overlaps with 5.	A7.1 Participants can/could involve themselves and others in the [intervention]<br>A7.2 Participants can/could buy in to the intervention and invest effort into its planning<br>A7.3 Participants can/could work together to operationalize the [intervention]	7. Participants can/could buy in to the [intervention] and invest effort into its planning	**7. Participants buy into the [intervention]**<br><br>**Enrolment:** The capacity and willingness of participants to organize themselves in order to collectively contribute to the work involved in the new practice.

8. Do/Did participants sustain their involvement in the [intervention]?<br><br>**Activation:** The capacity and willingness of participants to collectively define the actions and procedures needed to keep the new practice going.	(a) Does not emphasise problem of participants' sustaining support for the intervention;<br>(b) not clear what 'capacity' means or how it relates to involvement.	**A**8.1 Participants can/could continue to support the [intervention]	8. Participants can/could continue to support the [intervention]	**8. Participants continue to support the [intervention]**<br><br>**Activation:** The capacity and willingness of participants to collectively define the actions and procedures needed to keep the new practice going.

9. Can/Could participants' fulfill the tasks required of them by the [intervention]?<br><br>**Interactional Workability:** Whether people are able to enact the [intervention] and operationalise its components in practice	(a) Not clear whether this means doing the intervention in practice, or that people can fulfill the tasks, given their skill set and responsibility; (b) poor fit between explanation and statement .	**A**9.1 Participants' could/did perform the tasks required by the [intervention]<br>A9.2 The intervention improves/ improved interactions between participants, necessary to accomplish the work or tasks<br><br>**D9.1 Interactional Workability:** Whether people are able to enact the [intervention] and operationalise its components in practice<br>**D9.2 Interactional Workability:** Whether the intervention promotes or inhibits the congruence and disposal of work during interactions	9. Participants' could/did perform the tasks required by the [intervention]<br><br>**Interactional Workability**: Whether people are able to enact the [intervention] and operationalise its components in practice	**9. Participants' perform the tasks required by the [intervention]**<br><br>**Interactional Workability**: Whether people are able to enact the [intervention] and operationalise its components in practice

10. Can/Could participants' maintain confidence in the [intervention]?<br><br>**Relational Integration:** Whether people maintain trust in the [intervention] and in each other.	(a) insufficiently specific about what trust and confidence mean;<br>(b) not clear whether trust and confidence are different or same concepts;<br>(c) not clear whether people" in the description are the "participants" referred to I the statement<br>.	**A**10.1 Participants' could/did have confidence in the [intervention]<br>A10.2 The intervention improves\maintains the trust participants have in each other's work and expertise<br>**D10.1 Relational Integration:** Whether participants maintain confidence in their work when using the [intervention]<br>**D10.1 Relational Integration:** Whether the intervention improves accountability and confidence within networks involved	10. The intervention improves\maintains the trust participants have in each other's work and expertise<br><br>**Relational Integration**: Whether people maintain trust in the [intervention] and in each other.	**10. Participants maintain their trust in each other's work and expertise through the [intervention]**<br><br>**Relational Integration:** Whether people maintain trust in the [intervention] and in each other.

11. Is/Was the work of the [intervention] allocated to appropriate participants?<br><br>**Skill set Workability:** Whether the work required by the [intervention] is seen to be parcelled out to participants with the right mix of skills and training to do it.	N/A	**A**11.1 The work of the [intervention] is/was allocated to appropriate participants	11. The work of the [intervention] is/was allocated to appropriate participants	**11. The work of the [intervention] is appropriately allocated to participants**<br><br>**Skill set Workability:** Whether the work required by the [intervention] is seen to be parcelled out to participants with the right mix of skills and training to do it.

12. Is/Was the [intervention] adequately supported?<br><br>**Contextual Integration:** Whether the [intervention] is supported by management and other stakeholders, policy, money and material resources.	Statement does not make clear how or from whom support is forthcoming	**A**12.1 The [intervention] is/was adequately supported by its host organization	12. The [intervention] is/was adequately supported by its host organization	**12. The [intervention] is adequately supported by its host organization**<br><br>**Contextual Integration:** Whether the [intervention] is supported by management and other stakeholders, policy, money and material resources.

13. Can/Could participants discover the effects of the [intervention]?<br><br>**Systematization:** Whether participants can determine how effective and useful the [intervention] is from the use of formal and/or informal evaluation methods	(a) Not clear what 'discover' means in this context; (b) not clear that discover is the right word; (c) not clear how discovery is accomplished; (d) perceived overlap with 14 and 15.	**A**13.1 Participants have/had access to information about the effects of the [intervention]	13. Participants have/had access to information about the effects of the [intervention]	**13. Participants access information about the effects of the [intervention]**<br><br>**Systematization:** Whether participants can determine how effective and useful the [intervention] is from the use of formal and/or informal evaluation methods

14. Do/Did Participants share agreement about worth of the [intervention]'s effects?<br><br>**Communal appraisal:** Whether, as a result of formal monitoring, participants collectively agree about the worth of the effects of the [intervention]	(a) Difficult to distinguish between from statement 15 because it does not sufficiently emphasise *collective* appraisal;<br>(b) not clear whether this relates to 'worth' of the intervention or something else;<br>(c) not clear how this is different from statement 2, because it does not emphasise *appraisal* sufficiently.	**A**14.1 Participants could/did work together to assess the worth of the [intervention]'s effects<br>**A**14.2 Participants could/did collectively assess the [intervention] as worthwhile	14. Participants could/did collectively assess the [intervention] as worthwhile	**14. Participants collectively assess the [intervention] as worthwhile**<br><br>**Communal appraisal:** Whether, as a result of formal monitoring, participants collectively agree about the worth of the effects of the [intervention]

15. Do/Did participants personally attribute worth to the effects of the [intervention]?<br><br>**Individual appraisal:** Whether individuals involved with, or affected by, the [intervention], think it is worthwhile.	(a) difficult to distinguish between from statement 14 because it does not sufficiently emphasise *individual* appraisal;<br>(b) not clear how this is different from statement 3, because it does not emphasise *appraisal* sufficiently.	**A**15.1 Participants could/did individually assess the worth of the [intervention] effects<br>A15.2 Participants could/did individually assess the [intervention] as worthwhile	15. Participants could/did individually assess the [intervention] as worthwhile	**15. Participants individually assess the [intervention] as worthwhile**<br>**Individual appraisal:** Whether individuals involved with, or affected by, the [intervention], think it is worthwhile.
16. Can/Could participants modify their work in relation to the [intervention]?<br><br>**Reconfiguration:** Whether individuals or groups using the [intervention] can make changes as a	(a) the statement does not make clear that reconfiguration means modifying work in response to experience of implementing the intervention.<br>(b) the description suggests that this	**A**16.1 Participants could/did modify their work in response to their appraisal of the [intervention]	16. Participants could/did modify their work in response to their appraisal of the [intervention]	**16. Participants modify their work in response to their appraisal of the [intervention]**<br><br>**Reconfiguration:** Whether individuals or groups using the [intervention] can make changes as a

result of individual and communal appraisal.	concerns changes as a result of perceptions of the worth of the intervention.		result of individual and communal appraisal.
