# Supplementary material for: Evaluating complex interventions and health technologies using normalization process theory: development of a simplified approach and web-enabled toolkit
Source: BMC Health Serv Res. 2011 Sep 30;11:245. doi: 10.1186/1472-6963-11-245 (PMC3205031; doi:10.1186/1472-6963-11-245)
Supplement: Additional file 2 — Qualitative Data Collection Using On-Line. Pro Forma - Questions Asked. List of questions asked on online survey. [file 1472-6963-11-245-S2.PDF]

Additional File 2: Qualitative Data Collection Using On-Line Pro Forma – Questions Asked

1. Name and Institution
2. Please tell us about the implementation/integration problem you worked through using the 16-question NPT tool
3. What was your overall impression of the 16-question NPT tool?
4. In relation to the QUESTIONS, were there any particular questions that were difficult to answer or think with? Please explain why and/or outline any changes you would make.
5. In relation to the DESCRIPTIONS, were there any particular descriptions that were difficult to answer or think with? Please explain why and/or outline any changes you would make.
6. What could we do to improve the current version of the 16-question NPT tool?
7. How familiar are you with NPT?
8. Could you give us some context about how you used the 16-question NPT tool? For example: how long you worked with the 16-question NPT tool; whether you used it alone or with others; if with others, how familiar were they with NPT; did you consult specific resources to answer the questions or just from memory; and anything else you think might be useful.
